# Acute Presentation of Giant Hydronephrosis in an Adult

**DOI:** 10.7759/cureus.8702

**Published:** 2020-06-19

**Authors:** Meenu Joseph, Danny Darlington

**Affiliations:** 1 Nephrology, Pondicherry Institute of Medical Sciences, Pondicherry, IND; 2 Urology, Pondicherry Institute of Medical Sciences, Pondicherry, IND

**Keywords:** acute abdomen, giant hydronephrosis, ureteropelvic junction obstruction

## Abstract

Giant hydronephrosis is defined as a dilated collecting system containing more than one liter of fluid. The diagnosis of giant hydronephrosis is rare due to improved diagnostics and the liberal use of abdominal imaging. Herein we report a 40-year-old woman who presented with acute onset abdominal pain and was diagnosed with giant hydronephrosis. She underwent a simple open nephrectomy and made an unremarkable recovery. Although giant hydronephrosis due to ureteropelvic junction obstruction is common in the pediatric and adolescent age group, it rarely presents in adults. Acute presentations, like abdominal pain, are exceedingly rare. Judicial use of cross-sectional imaging, as in our patient, can confirm the diagnosis and help in successful management.

## Introduction

Idiopathic obstruction at the ureteropelvic junction (UPJ) is a major cause of obstructive uropathy at all ages and is often detected and treated in early childhood. It is a spectrum of pathophysiological processes of congenital (intrinsic stenosis, adynamic segment, mucosal valves and high insertion of the ureter) and acquired (scarring, reflux, malignant or benign tumors) etiologies [1]. The incidence of UPJ obstruction (UPJO) is less defined in the adult population with a male predominance of 2:1 and the left kidney being twice affected than the right. However, some cases remain silent and usually present in adulthood with vague abdominal pain associated with recurrent urinary tract infection or chronic back pain exacerbated by increased fluid intake, thereby mimicking gastrointestinal disorders [2]. Giant hydronephrosis, which implies a collecting system containing more than one liter of fluid, is becoming increasingly rare with the widespread availability of imaging facilities [3]. Congenital UPJO presenting in the fourth decade of life as an acute abdominal emergency is exceedingly rare. Here we report a case of giant hydronephrosis secondary to UPJO presenting emergently with acute abdominal pain.

## Case presentation

**Figure 1:**
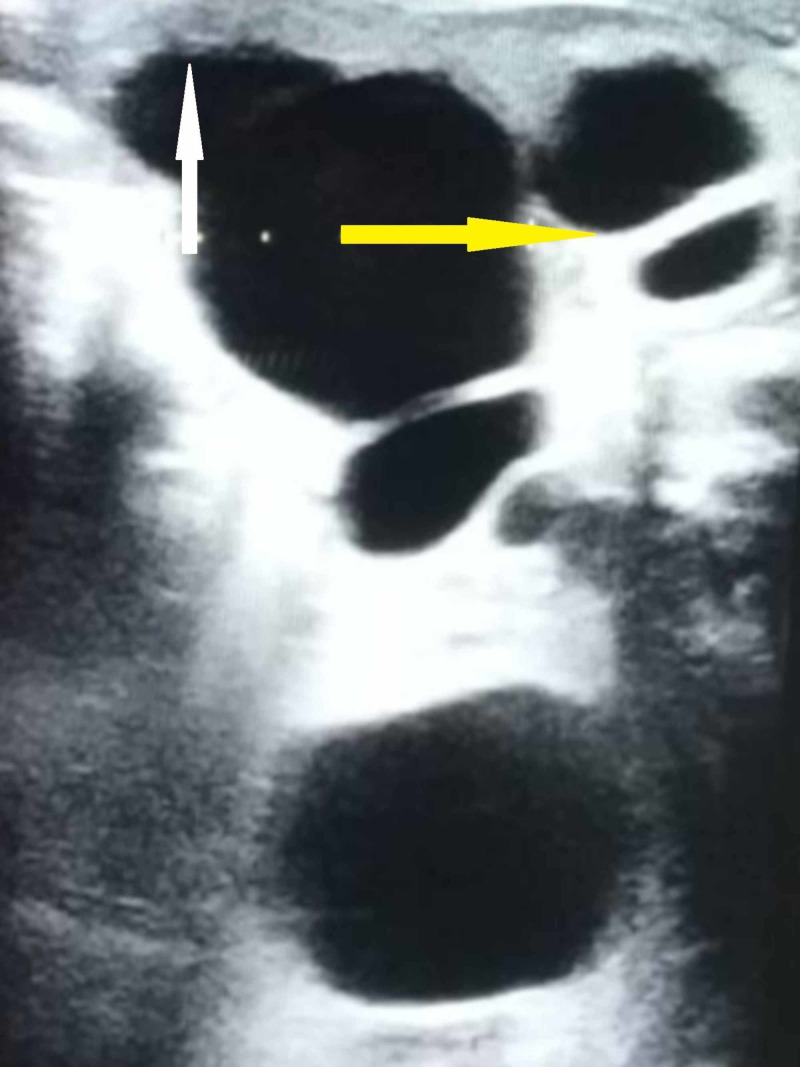
Ultrasonogram image depicting the grossly dilated lower calyx (yellow arrow) with a thin rim of renal parenchyma (white arrow)

A 40-year-old woman presented to the casualty of our tertiary care hospital with acute onset left loin pain and dyspnea of one-day duration. The pain was not radiating and was felt predominantly over the left loin and lumbar regions. There was no previous history of hematuria or urolithiasis. She denied any history of trauma or previous abdominal surgeries and did not suffer from any comorbid illness. She was afebrile, and the rest of her general examination was unremarkable. Vital signs like pulse rate and blood pressure were normal, while the abdominal examination revealed a localized bulge in the epigastric, umbilical, left lumbar, hypochondriac, iliac fossa, and renal angle regions on inspection. Palpation confirmed a tender, firm, vague mass that moved with respiration. The mass was bimanually palpable and ballotable clinically, indicating a renal mass. The renal angle was tender on palpation, but there was no other evidence of sepsis. Findings of an examination of the respiratory and cardiovascular system were normal. Her laboratory evaluation results, such as renal function tests, electrolytes, complete hemogram, urine microscopy, and urine culture, were within reference limits. Findings from her chest roentgenogram, electrocardiography, and echocardiography were also normal. An ultrasonogram of the abdomen revealed a 26 cm x 20 cm x 15 cm hydronephrotic left kidney pushing the bowel and other solid organs to the right side (Figure 1). The cortex was thinned out, measuring only 2 mm in thickness. However, the contralateral kidney was normal, and so were the urinary bladder, uterus, and ovaries.

**Figure 2:**
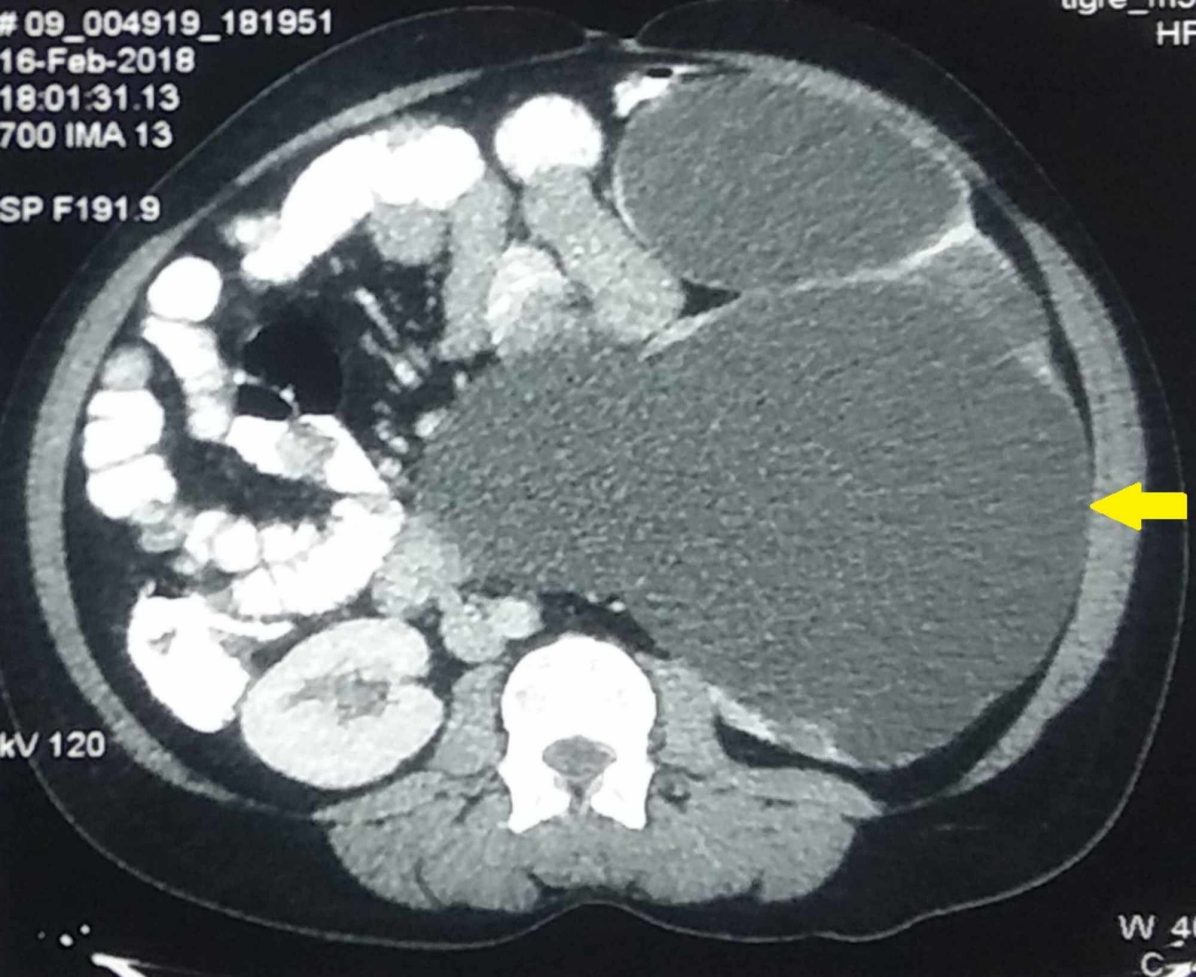
Contrast-enhanced computed tomography showing the giant hydronephrotic left kidney (arrow) without contrast excretion and the bowel being pushed to the contralateral side

Contrast-enhanced computed tomography (CECT) of the abdomen was done, which revealed a grossly enlarged left kidney (measuring 30 cm x 20 cm x 18 cm) with no uptake, excretion, or drainage of contrast into the collecting system (Figure 2). The ureter was normal in caliber, and the opposite kidney was normal. The patient was unwilling to undergo a percutaneous nephrostomy, and hence, a therapeutic aspiration of the giant hydronephrotic kidney was done under ultrasound guidance, which yielded clear fluid. 

**Figure 3:**
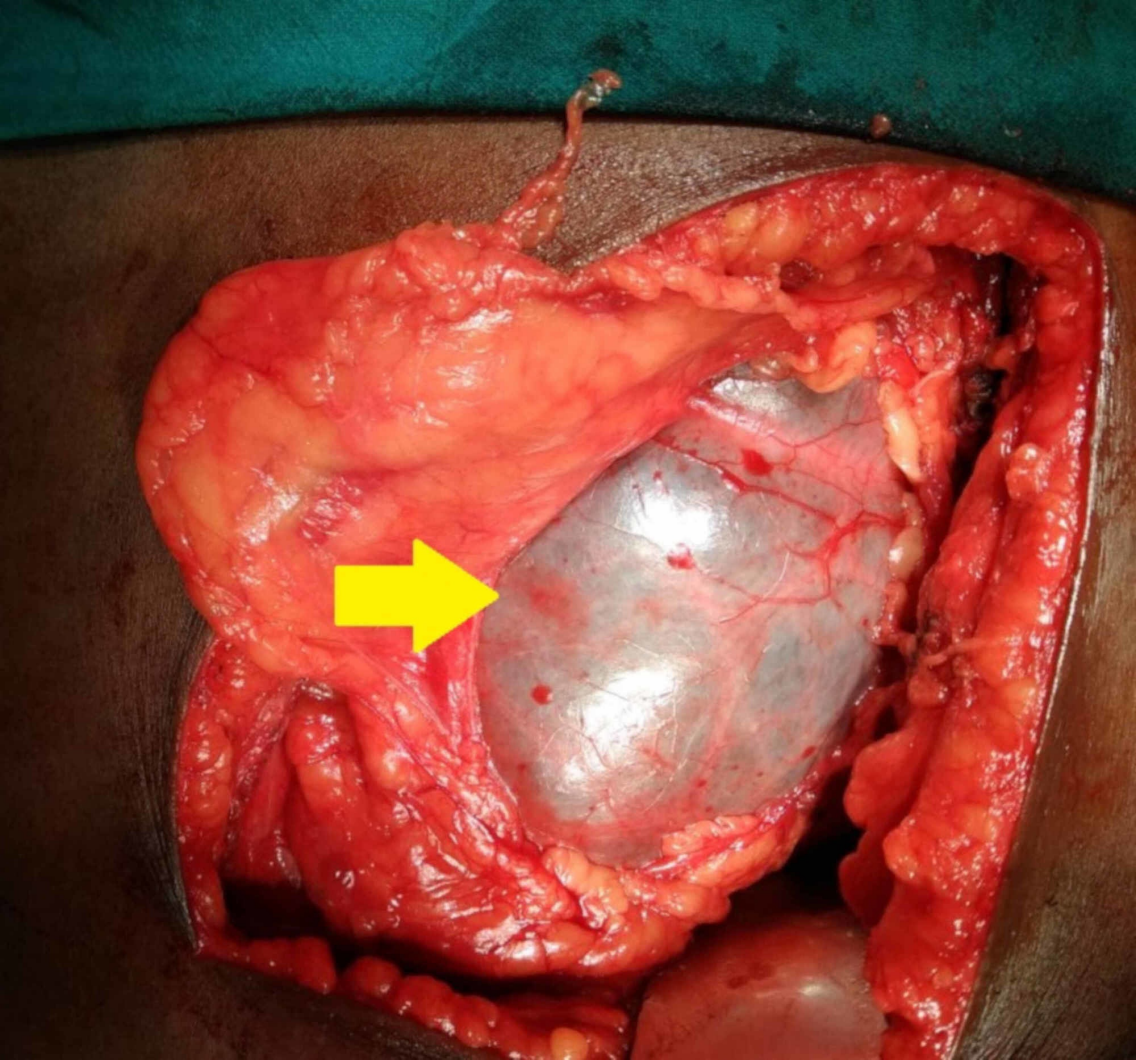
Operative image depicting the giant hydronephrosis with thinned out parenchyma (arrow)

**Figure 4:**
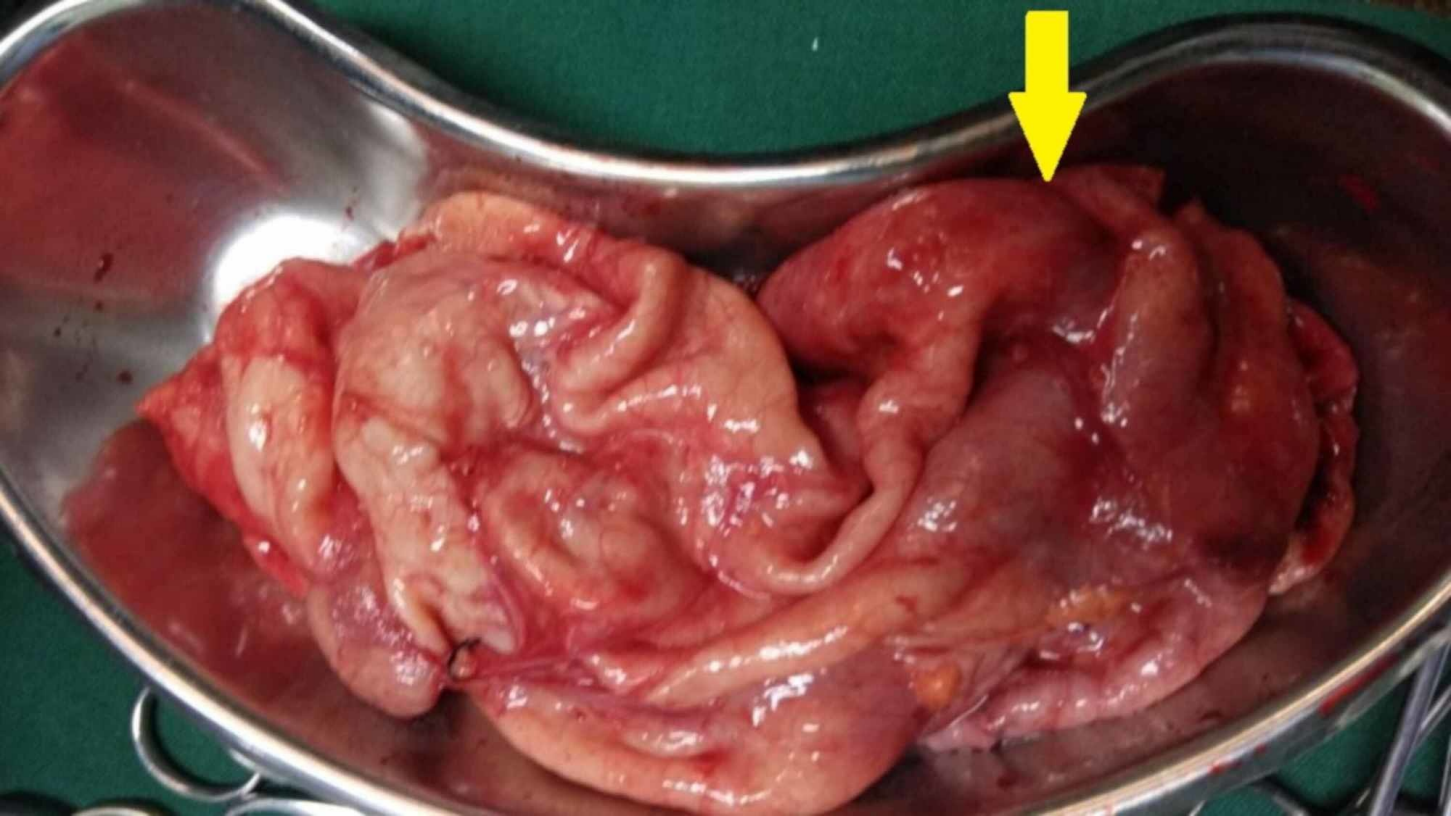
Operative nephrectomy specimen with baggy renal parenchyma (arrow)

In light of the giant hydronephrotic nonfunctioning left kidney, a left-sided open simple nephrectomy was performed through a left eleventh rib flank incision using the extrapleural and extraperitoneal approach after informed consent. The giant hydronephrosis crossed the midline pushing the bowel to the opposite side and reached inferiorly as far as into the pelvis (Figure 3). It was decompressed after exposing the kidney all around to access the renal hilum, and approximately 10 liters of clear fluid was drained. The renal parenchyma was thinned out and resembled a sac; however, the ureter was normal with abrupt narrowing at the level of UPJ (Figure 4). The renal artery was small and atretic, whereas the corresponding vein was dilated. They were ligated, thereby completing the nephrectomy, and the patient made an uneventful recovery.

## Discussion

Giant hydronephrosis is the presence of more than one liter of fluid in the collecting system of a hydronephrotic kidney. Although it is classically caused by UPJO, other conditions such as ovarian cysts, pancreatic cysts, hydatid cysts, and large adrenal cysts can mimic this peculiar condition of the kidney [4]. With the advent of imaging facilities and widespread abdominal imaging, hydronephrosis is picked up early in the course, and giant hydronephrosis is rarely diagnosed. Giant hydronephrosis is caused by UPJO, pelvic calculi, duplex systems with obstruction, and obstructive megaureter [5]. UPJO causing giant hydronephrosis is common among pediatric and adolescent patients [6]. The present case is rare, given that the patient is 40 years old and never had any previous symptoms except for the acute presentation.

Diagnosis of giant hydronephrosis is usually made preliminarily by ultrasonogram, which demonstrates the giant fluid-filled hydronephrotic sac and thinned out renal parenchyma. However, cross-sectional images and CECT with delayed images are needed to ascertain the functional status of the hydronephrotic kidney. Contrast uptake, excretion, and drainage in the collecting system can be assessed [7]. MRI is preferred in patients with deranged renal function. A diuretic renogram is needed to assess the differential function of kidneys and assess the drainage [2]. In our case, CECT was done, which confirmed the presence of giant nonfunctioning hydronephrotic kidney. The presence of giant hydronephrosis predisposes the patient to several complications. Hypertension, hematuria, traumatic perforation, and pyonephrosis have been reported in chronic hydronephrosis. Hence, giant hydronephrosis requires treatment, and trauma should be avoided while awaiting definitive surgery [8].

The treatment of giant hydronephrosis depends on the functional status of the kidney and underlying etiology. Nephrectomy is the procedure of choice for nonfunctioning kidneys, and pyeloplasty is the surgical management of UPJO with good differential function [9]. Percutaneous nephrostomy can be inserted as a temporizing procedure [10]. Other interesting endourological procedures, such as endopyelotomy, endopyeloplasty, and ureteric stenting, are used based on the patient's preference; however, their noninferiority compared to open and minimally invasive pyeloplasty is yet to be proven on large-scale studies [11]. The present case underwent open nephrectomy considering the nonfunctional status of the hydronephrotic kidney.

## Conclusions

This case has been presented for its rarity and to emphasize the fact that although UPJO is a rare diagnosis in adults presenting with an acute abdomen, it can still be detected as late as the fourth decade of life in developing and underdeveloped countries. Therefore, a high clinical suspicion is required for clinicians to diagnose this unexpected entity among adults with acute onset abdominal pain.

## References

[1] Ellerkamp V, Kurth RR, Schmid E, Zundel S, Warmann SW, Fuchs J (2016). Differences between intrinsic and extrinsic ureteropelvic junction obstruction related to crossing vessels: histology and functional analyses. World J Urol.

[2] Borin JF (2017). Ureteropelvic junction obstruction in adults. Rev Urol.

[3] Wang Q-F, Zeng G, Zhong L (2016). Giant hydronephrosis due to ureteropelvic junction obstruction: a rare case report, and a review of the literature. Mol Clin Oncol.

[4] Para SA, Wani SA, Murty K (2020). Giant hydronephrotic kidney in adolescence: a rare case report and a review of the literature. Urol Case Rep.

[5] Shah SA, Ranka P, Dodiya S, Jain R, Kadam G (2004). Giant hydronephrosis: What is the ideal treatment?. Indian J Urol.

[6] Kaura KS, Kumar M, Sokhal AK (2017). Giant hydronephrosis: still a reality!. Turk J Urol.

[7] Liao X-X, Yang J-H, Xing N-Z (2020). Intractable hiccup due to giant hydronephrosis: a rare case report and literature review. Int J Surg Case Rep.

[8] El-Atat R, Derouiche A, Slama MR, Chebil M (2011). Kidney trauma with underlying renal pathology: Is conservative management sufficient?. Saudi J Kidney Dis Transpl.

[9] Kausik S, Segura JW (2003). Surgical management of ureteropelvic junction obstruction in adults. Int Braz J Urol.

[10] Sharma U, Yadav S, Tomar V (2015). Factors influencing recoverability of renal function after urinary diversion through percutaneous nephrostomy. Urol Ann.

[11] Desai MM, Desai MR, Gill IS (2004). Endopyeloplasty versus endopyelotomy versus laparoscopic pyeloplasty for primary ureteropelvic junction obstruction. Urology.

